# Supplementary material for: Predicting visual function by interpreting a neuronal wiring diagram
Source: Nature. 2024 Oct 2;634(8032):113–23. doi: 10.1038/s41586-024-07953-5 (PMC11446822; doi:10.1038/s41586-024-07953-5)
Supplement: Supplementary file 6 — CRF and ERF predictions for individual TmY4 and TmY9 cells. Analogous to Supplementary Data 3, but for TmY target types. Shown are the top four monosynaptic pathways, the strongest pathway passing through each of the top ten intermediary types (ranking from Extended Data Fig. 7), and the trisynaptic pathway Tm1–TmY–Dm3–TmY (see the section entitled Prediction of spatial normalization). [file 41586_2024_7953_MOESM6_ESM.zip › DataS4/TmY9q/720575940616962491.pdf]

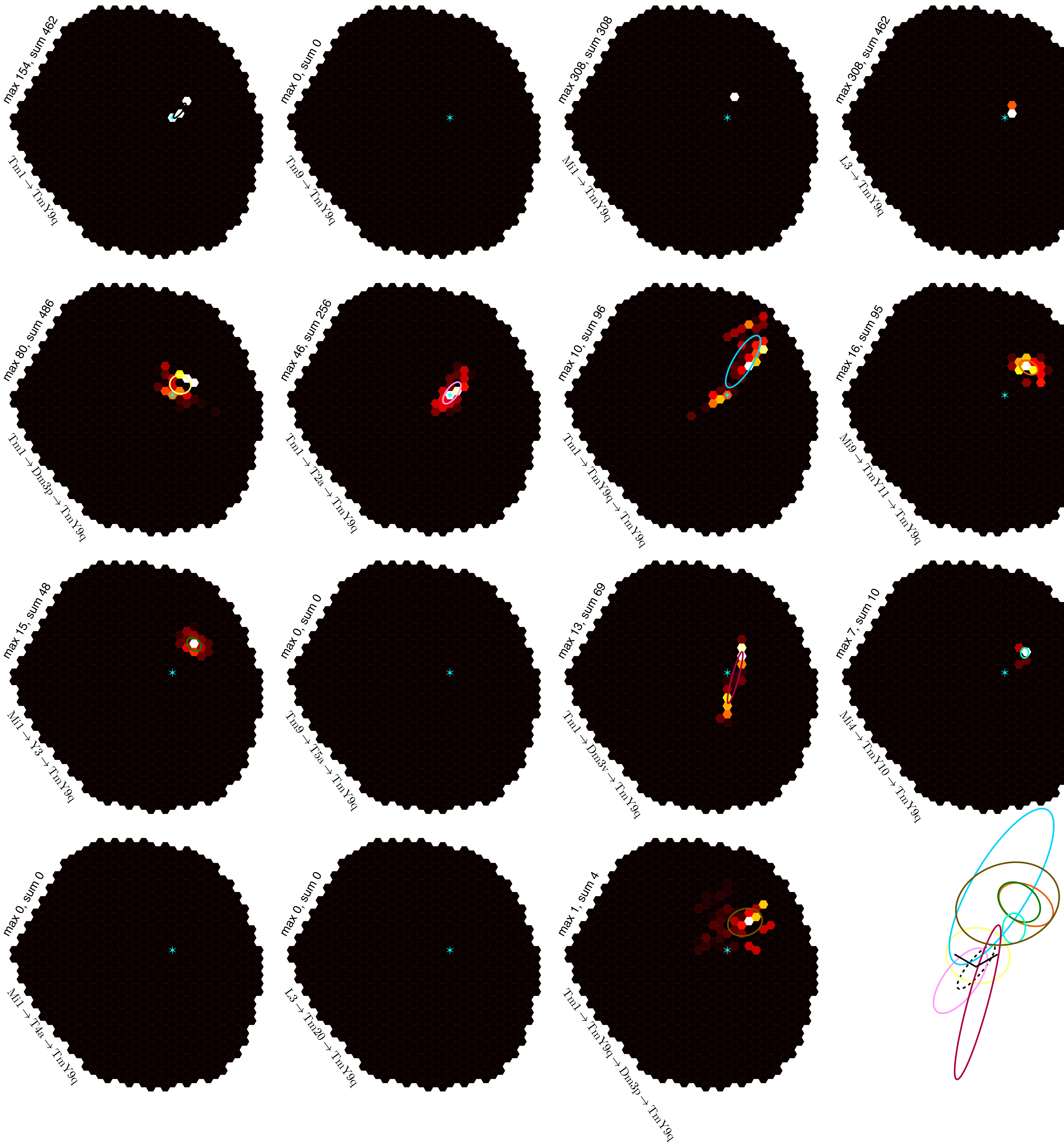
max 154, sum 462
Tm1 → TmY9q
max 0, sum 0
Tm9 → TmY9q
max 308, sum 308
Mi1 → TmY9q
max 308, sum 462
L3 → TmY9q
max 80, sum 486
Tm1 → Dm3p → TmY9q
max 46, sum 256
Tm1 → T2a → TmY9q
max 10, sum 96
Tm1 → TmY9q → TmY9q
max 16, sum 95
Mi9 → TmY11 → TmY9q
max 15, sum 48
Mi1 → Y3 → TmY9q
max 0, sum 0
Tm9 → T5a → TmY9q
max 13, sum 69
Tm1 → Dm3v → TmY9q
max 7, sum 10
Mi4 → TmY10 → TmY9q
max 0, sum 0
Mi1 → T4a → TmY9q
max 0, sum 0
L3 → Tm20 → TmY9q
max 1, sum 4
Tm1 → TmY9q → Dm3p → TmY9q
